# Microvesicles and intercellular communication in the context of parasitism

**DOI:** 10.3389/fcimb.2013.00049

**Published:** 2013-09-06

**Authors:** Natasha S. Barteneva, Natalia Maltsev, Ivan A. Vorobjev

**Affiliations:** ^1^Program in Cellular and Molecular Medicine, Children's Hospital Boston and Department of Pediatrics, Harvard Medical SchoolBoston, MA, USA; ^2^Department of Human Genetics, University of ChicagoChicago, IL, USA; ^3^A.N. Belozersky Institute of Physico-Chemical Biology, Lomonosov Moscow State UniversityMoscow, Russia; ^4^Department of Cell Biology and Histology, Faculty of Biology, Lomonosov Moscow State UniversityMoscow, Russia

**Keywords:** microvesicles, exosomes, miRNA, parasite, metabolism synchronization, horizontal gene transfer, co-infection, *Plasmodium*

## Abstract

There is a rapidly growing body of evidence that production of microvesicles (MVs) is a universal feature of cellular life. MVs can incorporate microRNA (miRNA), mRNA, mtDNA, DNA and retrotransposons, camouflage viruses/viral components from immune surveillance, and transfer cargo between cells. These properties make MVs an essential player in intercellular communication. Increasing evidence supports the notion that MVs can also act as long-distance vehicles for RNA molecules and participate in metabolic synchronization and reprogramming eukaryotic cells including stem and germinal cells. MV ability to carry on DNA and their general distribution makes them attractive candidates for horizontal gene transfer, particularly between multi-cellular organisms and their parasites; this suggests important implications for the co-evolution of parasites and their hosts. In this review, we provide current understanding of the roles played by MVs in intracellular pathogens and parasitic infections. We also discuss the possible role of MVs in co-infection and host shifting.

## Introduction

Production of membrane-enclosed microvesicles (MVs) is a universal feature of cellular life and has been demonstrated for organisms as diverse as Proteobacteria, Archaea, plants, and animals (Ellis and Kuehn, 2010; Silverman and Reiner, 2012; Deatherage and Cookson, 2012). Several distinct categories of membrane-enclosed MVs exist, including exosomes, ectosomes, and apoptotic bodies (in multi-cellular organisms). MVs are grouped based on their size, density, method of isolation, and markers, and purified MVs usually represent a mixture of aforementioned vesicular fractions.

Secretion of MVs is well-documented for prokaryotic and eukaryotic cells (György et al., 2011; Silverman and Reiner, 2012), and in infected organisms they can contain both host and parasitic antigens. Vesicles from a number of pathogens, such as *Leishmania, Cryptococcus*, and *Trypanosoma*, may carry on virulence factors and participate in their delivery to host cells (Silverman and Reiner, 2011, 2012; Lambertz et al., 2012; Torrecilhas et al., 2012), promoting dissemination of the pathogen.

Though it has become clear that MVs possess immunomodulatory features, little is known about the role of MVs in host-parasite co-existence and co-evolution. We anticipate that recent findings regarding the participation of MVs in the transfer of genetic information will expand the functions attributed to MVs in the host-parasite evolution. We will hypothesize about the role MVs play, as a vehicle for regulatory molecules important for synchronization of host and parasite metabolism, and for delivery of nucleic acids.

## Microvesicles are important intercellular communicators

MVs are considered a universal transport vehicle for intercellular communication. MVs incorporate peptides, proteins, lipids, miRNA, and mRNA, all of which can be transferred and become functional in target cells (Ratajczak et al., 2006; Valadi et al., 2007; Skog et al., 2008; Iglesias et al., 2012). MVs bind to cells through receptor-ligand interactions, fuse with target cell membranes, and deliver their cargo to the cytoplasm of the target cell. As has been observed with tumor-derived MVs, MVs can be enriched in specific coding and non-coding RNAs, chromosomal and mitochondrial DNA, retrotransposon RNA, and Alu transposon elements (Ronquist et al., 2009, 2012; Guescini et al., 2010; Balaj et al., 2011; Rak and Guha, 2012; Waldenstroem et al., 2012). Transfer of functional genetic information by MVs was initially shown in the experiments with a reporter mRNA encoding GFP (Deregibus et al., 2007), where intact RNA transcripts capable of serving as templates for protein translation were enriched in shed MVs (Li et al., 2012).

MVs are considered the major “miRNA transporter” between cells, since most extracellular miRNAs are found in vesicles (Gallo et al., 2012; Xu et al., 2013). miRNAs have been identified in helminthes and in protozoa possessing *Argonaute/Dicer* genes, while they are absent in protozoa lacking enzymes required for RNAi-based interference, such as *Plasmodium* spp and *Cryptosporidium* (Baum et al., 2009; Manzano-Roman and Siles-Lucas, 2012). MV-mediated export of miRNA is selective (Zhang et al., 2010a; Jaiswal et al., 2012; Vickers and Remaley, 2012). mRNA and miRNA packaged in vesicles appear to be more stable and resistant to RNAse digestion in the body fluids, due to the lipid membrane of MVs (Li et al., 2012; Vickers and Remaley, 2012).

During their release, MVs may incorporate components that are originally alien to the cell, such as proteins and nucleic acids that are transiently or constitutively expressed via plasmid or viral vector. Recently, it was shown that exogenous plant miRNA is present in human plasma and animal tissues. These results invoked the idea that miRNAs could regulate gene expression across the kingdoms (Kosaka and Ochiya, 2011; Zhang et al., 2012). We speculate that miRNA derived from the bacterial gut community may be packaged by epithelial cells into MVs and then delivered to different parts of the body, starting with the liver. A recent intriguing finding by two independent groups of the exogenous RNAs of different origin in human plasma samples (Bacteria and Archaea, Fungi, Plants—Wang et al., 2012; microbial RNA sequences—Semenov et al., 2012) supports this assertion.

Overall, MVs as vehicles for miRNA and other regulatory molecules, such as regulatory sequences of mRNA, may play important role in the synchronization of metabolism between the host and its parasites.

## Microvesicle proteomics

The production of MVs rises sharply in many parasitic and infectious diseases (Campos et al., 2010; Barteneva et al., 2013; Table 1). Proteins identified in these MVs were related to vesicle trafficking, signaling molecules and transmembrane small channels and transporters (Rodrigues et al., 2008; Silverman et al., 2010a). We recently showed that significant percentage of proteins identified in MVs during malaria infection belong to classical and alternative complement pathway, components of cytoskeleton, glycolysis and lipid transport (Mantel et al., 2013). Metabolic enzymes related to glycolysis constitute the largest protein family in excretory/secretory proteome of helminth *E.caproni* (Sotillo et al., 2010). Some glycolytic enzymes in parasitic MVs have separate function that make them important for parasite survival and dissemination (for example, binding of plasminogen for enolase in *Leishmania*) (Chandra et al., 2010). Furthermore, the MVs-production may explain the presence of atypical proteins lacking classical secretion signal peptides, like enolase, in the parasite secretions.

**Table 1:** **Microvesicles produced in response to different parasitic pathogens**.

Pathogen	Type of microvesicles (according to publication authors)	References
**FUNGI**		
*Cryptococcus neoformans*	Exosomes	Yoneda and Doering, 2006; Rodrigues et al., 2008; Nicola et al., 2009; Panepinto et al., 2009; Oliveira et al., 2010; Huang et al., 2012
*Malassezia sympodialis*	Exosomes	Gehrmann et al., 2011
*Paracoccidiodes*	Conditioned medium(secreted proteins and vesicles)	Weber et al., 2012
*Paracoccidiodes brasilensis*		Vallejo et al., 2011, 2012a,b
**PROTOZOA**		
*Giardia lamblia*	Secretory vesicles	Benchimol, 2004; Gottig et al., 2006
*Leishmania*	Exosomes from infected macrophages	Silverman et al., 2010a,b; Silverman and Reiner, 2011; Figuera et al., 2012; Hassani and Olivier, 2013
*Plasmodium vivax*	Plasma-derived MPs	Campos et al., 2010
*Plasmodium berghei*	Plasma-derived MPs (from infected mice)	Combes et al., 2005; Couper et al., 2010
*Plasmodium falciparum*	Vesicles(60–100 nm); microvesicles (100–1000 nm)	Trelka et al., 2000; Bhattacharjee et al., 2008; Mantel et al., 2013; Regev-Rudzki et al., 2013
*Plasmodium yoelii*	Plasma-derived exosomes	Martin-Jaular et al., 2011
*Toxoplasma gondii*	Exosomes	Bhatnagar et al., 2007
*Trypanosoma brucei*	Exosomes	Geiger et al., 2010
*Trypanosoma cruzi*	Outer membrane-derived vesicles, exosomes	Goncalves et al., 1991; Ouassi et al., 1992; Trocoli Torrecilhas et al., 2009; Cestari et al., 2012; Bayer-Santos et al., 2013
**MYCOPLASMA**		
*Mycoplasma*	Exosomes	Quah and O'Neill, 2007; Yang et al., 2012
**BACTERIA**		
*Borrelia burgdoferi*	Ectosomes (outer membrane vesicles)	Toledo et al., 2012
*Brucella abortus*	Ectosomes (outer membrane vesicles)	Pollak et al., 2012
*Chlamydia trachomatis*	Exosomes, outer membrane vesicles	Zhong, 2011; Frohlich et al., 2012
*Francisella novacida*		Pierson et al., 2011
*Legionella pneumophila*	Membrane vesicles	Galka et al., 2008
*Mycobacterium tuberculosis*	Exosomes; shedding microvesicles	Giri et al., 2010; Ramachandra et al., 2010; Singh et al., 2011, 2012; Duarte et al., 2012
*Mycobacterum avium*	Exosomes	Bhatnagar and Schorey, 2007
*Mycobacterium bovis*	Exosomes	Giri and Schorey, 2008
*Salmonella thyphimurium*	Outer membrane-derived vesicles	Yoon et al., 2011
**HELMINTHS**		
*Caernorhabditis elegans*	Exosomes	Liegeois et al., 2006
*Echinostoma caproni*	Exosomes	Andresen et al., 1989; Marcilla et al., 2012
*Echinococcus multilocularis*	Vesicles derived from metacestodes	Eger et al., 2003; Walker et al., 2004; Huebner et al., 2006; Nono et al., 2012
*Fasciola hepatica*	Exosomes	Marcilla et al., 2012

Parasite-induced MVs also contain constituent host proteins different depending on the species of parasite. For example, while mucin-2 was found in *E. caproni* vesicles, only CD19 and the constant region of the IgA heavy chain were found in *Fasciola hepatica* vesicles (Wilson et al., 2011; Marcilla et al., 2012). Conversely, the same helminth species when develops in several intermediate hosts exhibit host adaptation via differential expression of certain gene families (example: antigen B gene family from *E. granulosa*) in subsequent life cycle stages (Mamuti et al., 2007; Zhang et al., 2010b), however, no parasite proteomes from MVs produced in different intermediate hosts are currently available. MVs production increased during different developmental stages of parasites and proportion of specific antigens may be changed [as shown for RESA-antigen, during ring-stage, trophozoite and shizont stages of *P. falciparum* development (Natakamol et al., 2011)].

In sum, extracellular MVs contain parasite-specific excretory/secretory proteins (Silverman et al., 2010a; Marcilla et al., 2012), often lacking signal sequences (*Leishmania*), and participate in delivery of virulence factors and regulation of parasite virulence (Silverman and Reiner, 2011; Torrecilhas et al., 2012). Majority of proteome studies of parasite-produced MVs identified virulence factors in the MVs proteomes (Geiger et al., 2010; Silverman et al., 2010a; Bayer-Santos et al., 2013). MVs deliver virulence factors such as toxins, proteases, adhesins (Amano et al., 2010; Torrecilhas et al., 2012), *Entamoeba histolytica* rhomboid protease (EhROM1) (Baxt et al., 2008), participate in regulation of gene expression and help to escape immune evasion (Lambertz et al., 2012).

## Immunomodulatory activities of microvesicles and molecular mimicry

Parasites have developed many strategies that support their transmission and allow them to survive and reproduce, such as development of novel cellular pathways that enable invasion into different hosts and diverse immune evasion strategies including: alteration of host antigens, establishment of self-tolerance, functional immune inactivation, immunosuppression, molecular mimicry between parasite polypeptides and host antigens, acquisition of sialic acid motifs from host cells and adsorption of host serum sialoglycoconjugates leading to the modulation of NETosis (Hahn et al., 2013), and antigenic variability regulated by parasite methyltransferases (Figueiredo et al., 2008). Production of MVs appears to be involved in many of these processes.

Molecular mimicry as a strategy for host manipulation and evasion of immune response is well-known in viruses because of their ability to acquire host proteins or genetic material during virion assembly (Alcami, 2003; Bernet et al., 2003). There is significant evidence that autoimmune disease can develop after bacterial or parasitic infection, such as Chagas disease, where a cross-reaction between cardiac muscle cells and *T. cruzi* occurs (Acosta and Santos-Buch, 1985; Sepulveda et al., 2000). Recently, molecular mimicry between a family of peptides produced by trematode helminthes, and human defense peptides, including defensins and cathelidicins was found (Robinson et al., 2011). This family of helminth defense molecules (HDMs) is conserved throughout trematodes, and these proteins participate in the host immune response modulation and anti-inflammatory action (Robinson et al., 2011).

Infections caused by intracellular pathogens and parasites are often chronic and lead to significant immunomodulation of host immune response by the parasite. MVs produced during protozoan infections were shown to participate in this process (Bhatnagar and Schorey, 2007; Bhatnagar et al., 2007; Barreto et al., 2010; Silverman et al., 2010a; Hassani and Olivier, 2013). For example, during *Plasmodium* infection, there are increased quantities of MVs in plasma, and they contain a significant amount of parasite material. These MVs induce neutrophil activation (Mantel et al., 2013) and strong pro-inflammatory activation of macrophages as measured by CD40 and TNF up-regulation (Couper et al., 2010). Besides, vesiculation, which utilizes host cell machinery, is an important mechanism for parasite egress in the case of *P. falciparum*, the cause of malaria and a member of the phylum *Apicomplexa* (Lew, 2011). Secreted vesicles, which in the case of helminthes, present among other parasite secretion products, have been shown to modulate host immune responses and strongly influence the outcome of infections to the parasite's advantage (Spolski et al., 2000; Allen and MacDonald, 1998; Silverman et al., 2010b).

**Figure 1:**
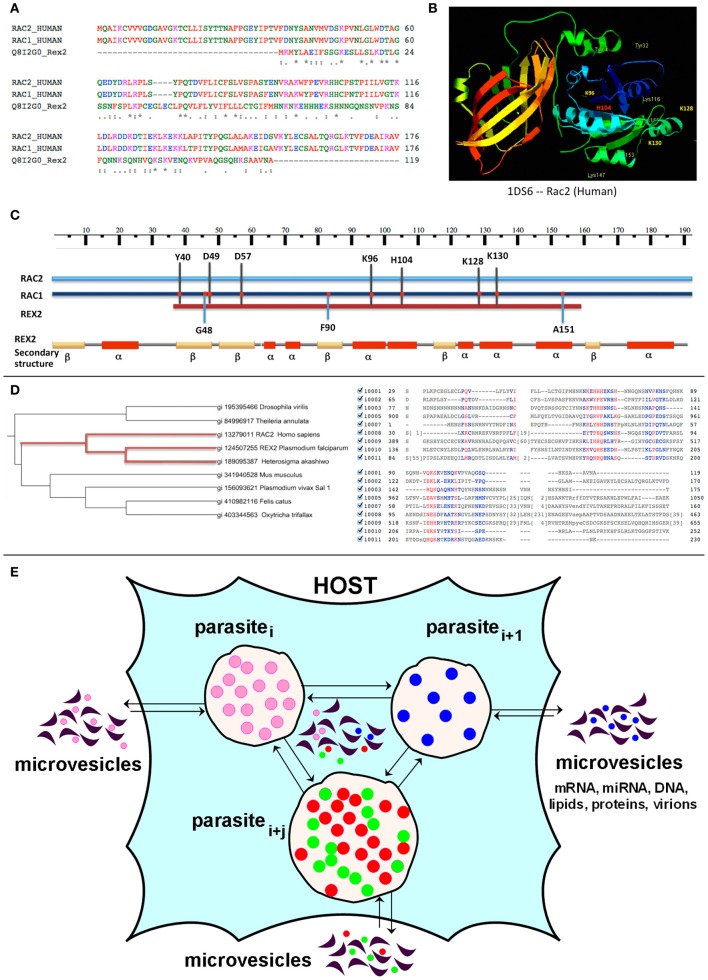
**Alignment and PSI-Blast analysis of Rex2 *P. falciparum* protein. (A)** ClustalW sequence alignment of Rex2 *P. falciparum* protein (NCBI accession XP_001352224; Uniprot ID Q8I2GO_PLAF7) with Human Rac1 protein (NCBI accession AAH04247; Uniprot ID RAC1_HUMAN) and Ras-related C3 botulinum toxin substrate 2 RAC2_HUMAN. As follows from the alignment **(A)** the N-terminus of the *P. falciparum* Rex2 protein shares significant similarity with the Rac1 and Rac2 proteins. In the active GTP-bound state these proteins regulate a variety of cellular responses, such as secretory processes, phagocytosis of apoptotic cells, and epithelial cell polarization. Rac2 activity also includes regulation of human neutrophil NADPH oxidase and activation of the production of reactive oxygen species (ROS). **(B)** Three-dimensional structure of human Rac2 (critical aminoacids are shown with numbers). **(C)** Sequence alignment between *P. falciparum* Rex2 and Rac2 and Rac1 proteins reveals an exact match in a number of functionally important amino acids positions, including (a) Asp57. RAC2 Asp57Asn mutation has been shown to be associated with severely impaired fMLP- or IL-8–induced neutrophil responsiveness, including adhesion, chemotaxis, and superoxide production. The Asp57-mutant Rac2 does not bind GTP and was found to act in a dominant-negative fashion for both Rac1 and Rac2 because of its tight GEF binding; (b) H103, which is involved in Rac1-mediated oxidase activation, and (c) the ubiquitination sites K96 and K123. The alignment also revealed exact matches found exclusively between the Rac1 and Rex2 proteins, including G48, F90 and A151. However, the functional impact of these amino acids is not yet known. **(D)** PSI-Blast analysis of *Plasmodium falciparum* 3D7 Rex2 protein against NCBI non-redundant database showed weak similarities to hypothetical proteins from parasitic *Apicomplexa Theileria annulata*, ciliate *Protozoa Oxytricha trifallax*, *Felis catus* (gi410982116), *Drosophila virilis* (gi195395466), as well as mouse Nrde2 protein (gi|19344080). Multiple sequence alignment of these proteins was developed using NCBI Cobalt (Papadopoulos and Agarwala, 2007). Maximum likelihood phylogenetic tree was developed using the iTOL server and default parameters (Letunic and Bork, 2011). As it follows from the tree the Rex2 protein most closely evolutionary relates to a hypothetical protein from algae *Heterosigma akashiwo* and Human Rac2 protein. Evolutionary relations between algae and *Apicomplexa* are well-established (Lemgruber et al., 2013), however, relatedness to Human Rac2 protein suggests HGT from parasite to Human. **(E)** A hypothetical scheme of MVs exchange in parasite-host interaction. Host and multiple parasites produce and exchange microvesicles, which transfer lipids, proteins, nucleic acids such as miRNA, mRNA, DNA, and may camouflage virions.

In our recent publication more than thirty parasite proteins in MVs derived from red blood cells infected with 3D7 or CS2 strains of *P. falciparum* were identified (Mantel et al., 2013). A modified approach, first described by Ludin et al. (2011) was employed, allowing for rigorous analysis of *P. falciparum* proteins that may potentially contribute to the infectious process via molecular mimicry of host molecules. Identified potential candidates include erythrocyte-binding proteins 1, 2, and 3, liver-stage antigen, and others (e.g., Rex2) (manuscript in preparation). Figures 1A–C shows the similarity between the *P. falciparum* short (119 amino acids) PEXEL (*Plasmodium* export element)-negative ring-exported protein 2 (Rex2) and the *H. sapiens* Rac1 and Rac2 proteins, providing one example of possible molecular mimicry and parasite-human HGT in parasitic invasion (Figure 1D). It was previously demonstrated (Haase et al., 2009) that a short sequence in the N-terminus and transmembrane domain of the Rex2 protein are both required for parasite export. The N-terminus of Rex2 exhibits significant similarity to the human small GTP-ases Rac1 and Rac2. A number of studies have demonstrated that deleterious mutations in Rac2 lead to defective chemotaxis, impaired phagocytosis, and decreased pathogen killing by macrophages and/or neutrophils (Roberts et al., 1999; Koh et al., 2005; Yamauchi et al., 2005; Zhang et al., 2009). Because it has been reported that neutrophils from malaria patients have reduced chemotactic activity (Nielsen et al., 1986; Leoratti et al., 2012), a role for Rex2 in molecular mimicry of Rac2 is likely. It is anticipated that *in silico* analysis of other pathogen-derived MV-associated proteins will be helpful in further understanding how MVs function as intercellular communicators during disease states, and provide insights on what to base future experimental studies on.

Molecular mimicry may be a more prevalent parasitic strategy than was previously thought (Ludin et al., 2011). Acquisition of complete nucleotide sequences or sequence motifs from the host may happen at different stages of parasite-host co-existence, and MVs may play a significant role in this molecular exchange.

## Do microvesicles participate in co-infection?

Parasitic and symbiotic associations are ubiquitous and often life-long relationships (Eckburg et al., 2005; Weiss and Aksoy, 2011). Every mammal possesses complex microbial communities that reside on all mucosal surfaces. The human gastrointestinal tract harbors an estimated 1014 species of microbes from over 500 distinct microbial taxa (Eckburg et al., 2005). Although infectious parasite biology research is still dominated by studies of single infections poly-parasitism is very common in nature (Petney and Andrews, 1998; Bordes and Morand, 2009). Infection with one parasitic species can have a large impact on host susceptibility to secondary infection, and this phenomenon is partially dependent on the duration of infection (Telfer et al., 2010).

There is a growing body of evidence that concurrent parasitic infections can confer benefits to their hosts. For example, *Wolbachia*, which is considered a reproductive parasite in arthropods (Werren et al., 2008), can provide metabolic advantages to their hosts during stressful conditions, such as increased haem and riboflavin availability (Brownlie et al., 2009; Hosokawa et al., 2010). There are a number of studies outlining associations between different parasitic co-infections. Thus, helminth infection can prevent or suppress autoimmune and allergic diseases depending on helminth burden (reviewed in Zaccone and Cooke, 2013). A similar finding has been described for malaria infection, where the disease can be asymptomatic or less severe, with concomitant lower parasitaemia, in helminth-infected patients (Adegnica and Kremsner, 2012). The infection of *Schistosoma haematobium* is associated with protection against acute *P. falciparum* infection (Lyke et al., 2005).

The role of MVs as messengers between parasite and host immune cells is well-established (Silverman and Reiner, 2012). We have shown involvement of MVs in cross-communication within a *P. falciparum* population (Mantel et al., 2013). The exchange of MVs derived from different parasites as well as from host cells can participate in the mechanisms of co-infection (Figure 1E). However, there are currently no published reports describing the communication of MVs derived from different parasites and the issue deserves in-depth elucidation.

## Searching for new clues in intercellular communication by *in silico* genomics and proteomics

Amoebae, as well as other free-living protozoan hosts for bacteria, fungi, giant DNA viruses and virophages are “melting pots” for HGT exchanges (Hotopp et al., 2007; Moliner et al., 2010; Raoult and Boyer, 2010; Lamrabet et al., 2012). Besides, DNA exchange may also occur in reverse from microorganisms to protozoa (Ricard et al., 2006), and to animals (McNulty et al., 2010; Dunning Hotopp, 2011). Examples of gene transfer from the animal host to the ancestor of the apicomplexan parasites include genes encoding proteins involved in cell adhesion, O-linked glycosylation and a major epigenetic regulator histone methyltransferase Set8 (Kishore et al., 2013). In many instances, interdomain HGT involves transfers between endosymbiotic bacteria and their hosts and from bacteria to asexual animals (Dunning Hotopp, 2011). For example, *Wolbachia*-to-arthropod HGT has been seen in the genomes of the bean beetle (Coleoptera) (Kondo et al., 2002), mosquitoes (Diptera) (Klasson et al., 2009; Woolfit et al., 2009), and other arthropods, as well as HGT in the opposite direction—from arthropod to *Wolbachia* (Duplouy et al., 2013). Some *Microsporidia* species acquired a gene from arthropods that encodes purine nucleotide phosphatase, though most HGT to *Microsporidia* identified to date derives from prokaryotes (Selman and Corradi, 2011).

Analysis of *T. cruzi* ribosomal proteins *in silico* identified significant homology not only with members of the animal kingdom (*H. sapiens, C. elegans, D. melanogaster*), but also with plants and protozoa (Wayengera, 2009). Recent massive bioinformatics analyses of whole genome sequences have shown that many intracellular prokaryotes have the ability to manipulate the eukaryotic ubiquitin system through molecular mimicry of the F-box component of the SCF E3-ubiquitin ligase eukaryotic-like F-box proteins (Price et al., 2009; Price and Kwaik, 2010).

The ability of MVs to serve as vehicles not only for proteins and lipids, but also for nucleic acids (Ronquist et al., 2009, 2012; Guescini et al., 2010; Balaj et al., 2011; Pisetsky et al., 2011; Rak and Guha, 2012), make us hypothesize that MV production and exchange are an important mechanisms in gene information exchange between parasites and their hosts. As described above, this is indirectly confirmed by a number of similarities between parasite protein sequences and host molecules. Recent studies of mammalian (equine) ovarian follicles revealed that intercellular communication in the ovarian follicle may involve transfer of miRNA and other bioactive molecules by MVs between follicular fluid and granulosa cells (da Silveira et al., 2012). In addition, the transfer of chromosomal DNA fragments by prostate-derived MVs to human sperm (vertical transfer) was described by Ronquist et al. (2009). Moreover, MVs camouflage viruses from immune surveillance and facilitate their access to cells (Kadiu et al., 2012).

Thus, we hypothesize that one of the important functions of MVs in parasite-host and parasite-parasite co-evolution is their participation in HGT. This hypothesis is based on the following properties of MVs: (1) the ability to transfer nucleic acids, including DNA; (2) the capability of MVs to protect and deliver genetic information to different organs, including reproductive organs in the case of multi-cellular organisms; and (3) the ability to transfer cargo to alien cells and tissues (in the case of parasites). Regev-Rudzki et al. (2013) recently provided additional support to this hypothesis confirming that *P. falciparum* derived MVs are capable of delivering genes between parasite populations inside their host.

## Concluding remarks

MVs are emerging as critical players in HGT including small non-coding RNAs. Major challenges in the field of extracellular vesicle research include (1) development of new comprehensive methods for their isolation and characterization, and (2) isolation of pure populations of specific MVs. Improved understanding of the mechanisms involved in vesicle shuttling of genetic information and proteins is crucial in order for new diagnostic and therapeutic strategies to be designed and implemented.

Dissemination of parasite components with MVs provides (1) a unique advantage in protection against host-mediated immune responses and nucleases from blood and other body fluids, (2) the possibility of reaching distant regions and evading immune attacks due to their small size (<1 μm), and (3) the capability of transferring genetic information long-distance—this may lead to direct participation of pathogenic components in the regulation of gene expression in the different host cells and metabolism synchronization between host and parasite, as well as HGT. This may contribute to further co-adaptation and co-evolution of the parasite and its host. Remarkably, MV exchange may happen similarly in dramatically different animal host cells, as well as in simple multicellular organisms. This can lead to broader host ranges and an increase in the virulence of certain parasites.

Our present knowledge of MVs derived from parasites comes from studies involving limited numbers of parasites and hosts. In the future our understanding of how parasite-host exchange of regulatory molecules and genetic information happens may change, especially when taking into account interactions between multiple parasitic species within the same host organism.

### Conflict of interest statement

The authors declare that the research was conducted in the absence of any commercial or financial relationships that could be construed as a potential conflict of interest.

## Abbreviations

7-AAD: 7-aminoactinomycin D
EAE: experimental allergic encephalomyelitis
EGFP: enhanced green fluorescent protein
GFP: green fluorescent protein
HDM: helminth defense molecules
HDP: host defense peptide
HGT: horizontal gene transfer
LDLRAP1 protein: low density lipoprotein receptor adapter protein
MAPK/ERK pathway: mitogen-activated protein kinases/Extracellular signal-regulated kinases pathway
miRNA: microRNA
mtDNA: mitochondrial DNA
MV: microvesicles
NETs: neutrophil extracellular traps
NETosis: *in vivo* NETs are released during a form of pathogen-induced cell death, which was recently named NETosis
ORF: open reading frame
PV: parasitophorous vacuole
PVM: parasitophorous vacuole membrane
ROS: reactive oxygen species.
